# Canine Adenovirus Type 2 Vector Generation via I-Sce1-Mediated Intracellular Genome Release

**DOI:** 10.1371/journal.pone.0071032

**Published:** 2013-08-01

**Authors:** Sandy Ibanes, Eric J. Kremer

**Affiliations:** 1 Institut de Génétique Moléculaire de Montpellier, CNRS, Montpellier, France; 2 Université de Montpellier I, Montpellier, France; 3 Université Montpellier II, Montpellier, France; University Claude Bernard Lyon 1, France

## Abstract

When canine adenovirus type 2 (CAdV-2, or also commonly referred to as CAV-2) vectors are injected into the brain parenchyma they preferentially transduce neurons, are capable of efficient axonal transport to afferent regions, and allow transgene expression for at last >1 yr. Yet, translating these data into a user-friendly vector platform has been limited because CAV-2 vector generation is challenging. Generation of E1-deleted adenovirus vectors often requires transfection of linear DNA fragments of >30 kb containing the vector genome into an E1-transcomplementing cell line. In contrast to human adenovirus type 5 vector generation, CAV-2 vector generation is less efficient due, in part, to a reduced ability to initiate replication and poor transfectibility of canine cells with large, linear DNA fragments. To improve CAV-2 vector generation, we generated an E1-transcomplementing cell line expressing the estrogen receptor (ER) fused to I-SceI, a yeast meganuclease, and plasmids containing the I-SceI recognition sites flanking the CAV-2 vector genome. Using transfection of supercoiled plasmid and intracellular genome release via 4-OH-tamoxifen-induced nuclear translocation of I-SceI, we improved CAV-2 vector titers 1,000 fold, and in turn increased the efficacy of CAV-2 vector generation.

## Introduction

Since the advent of human adenovirus (HAdV) vectors in the mid-1980’s, their use has expanded to address questions in almost all realms of applied and fundamental biology. In the early 1990’s, recombinant vectors were generated via homologous recombination (HR) in cell lines [1]. HAdV type 5 (HAdV5) E1 region-transcomplementing cells (HEK 293 cells), generated by Graham and van der Ebb [2], can be readily transfected and were used to produce HAdV ΔE1 vectors. Recombinant vector clones were isolated as individual plaques in cell monolayers overlaid with agarose, serially amplified and/or eventually screened by transgene expression and restriction digests of semi-purified vector DNA. To circumvent the generation and cloning in cell lines, and the time involved in growing and screening plaques, Ketner et al cloned HAdV genomes in plasmids using HR in S. *cerevisiae* [3]. Later Chartier et al. developed an approach where HAdV genomes could be generated using HR in E. *coli* [4]. Using these approaches the adenovirus genome is released by restriction enzyme(s) at sites flanking the inverted terminal repeats (ITRs), and then the DNA is transfected into cells to generate the vectors.

In the early 1990’s we initiated the generation of canine adenovirus type 2 (CAdV-2, or also commonly referred to as CAV-2) vectors [5], [6], [7], [8], [9]. One *raison d’être* was that vectors derived from non-primate adenoviruses may have characteristics that would circumvent the ubiquitous pre-existing humoral and cellular immunity in humans. For ∼7 years we were unable to generate homogeneous ΔE1 CAV-2 vector preparations [5]. Canine cell lines are notoriously difficult to transfect with linear DNA, which precluded efficient HR in the cells. A breakthrough for CAV-2 vector cloning and generation came when we adapted the protocol from Chartier et al. to clone a recombinant CAV-2 vector genome in a plasmid [10]. Yet, although we have optimized conditions, CAV-2 vector generation from cloned genomes remains labor-intense and often unproductive. For example, when we were able to generate a vector it was following the transfection of ∼10^7^ E1-transcomplementing cells, at an efficiency of ∼5%, we routinely generated <20 infectious particles. Compared to the titre of human HAdV5 vectors using the analogous approach, the titre produced after CAV-2 transfection is 10^4^- to 10^5^-fold lower. Importantly though, once a ΔE1 CAV-2 vector was made its propagation in CAV-2 E1-transcomplementing cells equaled that of HAdV5 vectors in 293 cells [11], arguing against a lack of efficient E1 trans-complementation [10].

CAV-2 vectors have a niche in fundamental and applied neuroscience due to the preferential transduction of neurons in the brains of rodents, dogs, and primates [12], [13], [14], [15], [16], [17], [18]. CAV-2 axonal transport can also be >100-fold more efficient than HAdV type 5 (HAdV5) vectors and lentivirus vectors [19], [20]. The tropism and axonal transport is likely due to the restricted use of the coxsackievirus adenovirus receptor (CAR), which is expressed by neurons in the brain parenchyma, and by transport in pH-neutral/RAb7^+^/CAR^+^ vesicles, respectively [19]. CAV-2 transduced neurons can also express a transgene for at least 1 yr in vivo [15], [16]. Combining these characteristics with a 30-kb cloning capacity in helper-dependent (HD) CAV-2 vectors makes them powerful tools to understand fundamental neurobiology. In addition, the paucity of crossreacting humoral and cellular immunity, and the inability of CAV-2 vector to induce human dendritic cell maturation suggest that CAV-2 vectors may be clinically relevant in some paradigms [21], [22], [23]. While gene transfer offers substantial potential to understand, prevent and treat neurodegenerative diseases, this strategy also has unique preclinical and clinical obstacles - in particular the need to test vector efficacy and safety in healthy and diseased paradigms [24].

Analogous to the challenges that adeno-associated virus (AAV) vectors faced in the mid-1990’s, a more user-friendly protocol to generate CAV-2 vectors will significantly advance their preclinical and clinical evaluation and use for more fundamental neurobiology questions. In this study, our goal was to eliminate the bottleneck for CAV-2 vector generation. We generated CAV-2 E1-transcomplementing cells line expressing the estrogen receptor (ER) fused to I-SceI, a yeast meganuclease, and plasmids containing the I-SceI recognition sites flanking the vector genome. Using transfection of supercoiled plasmid and intracellular vector genome release via 4-OH-tamoxifen (OHTam)-induced nuclear translocation of ER-I-SceI, we improved CAV-2 vector generation and reduced the time needed to produce a purified, high titered preparation. We also tried to further increase efficacy by dampening the DNA damage response by I-SceI-induced generation of free DNA ends.

## Materials and Methods

### Cell Line Generation

pBabe-I-SceI-ER has been previously described [25]. The MLV-ER-I-SceI-HA vector was made using standard protocols by cotransfecting pBabe-I-SceI-ER, a plasmid expressing GAG/Pol and the spike glycoprotein of the vesicular stomatitis virus (VSV-G) in 293 cells. The supernatant containing MLV-ER-I-SceI-HA was collected 36 h posttransfection and incubated with DK cells expressing the CAV-2 E1 region [26] for 24 h. The medium was then replaced with fresh medium containing 1 µg/ml of puromycin (STE Cayla) for 7 days and a polyclonal population of cells, referred to from herein as DKSce, was banked.

### CAV-2 Vector Generation

DKSce cells in 30 mm wells were incubated with 4 µg of DNA complexed with 8 µl of Turbofect/10^6^ cells. Three hours posttransfection, 300 nM of 4-OH-tamoxifen (OHTam) was added to the medium. One hour later, the medium was replaced with DMEM/10% FCS and the cells were kept at 37°C/5% CO_2_. On day 2, 3, 4, or 5, the cells were collected by scraping, lysed by three freeze/thaw cycles, the cellular debris pelleted by centrifugation [10] and the supernatant incubated with a fresh monolayer of 5×10^6^ DKZeo cells in a 10 cm plate for 24 h. Efficacy was assayed by fluorescent microscopy and/or flow cytometry.

### Plasmid Generation and Antibodies

Using unique NotI and AscI restriction enzyme sites flanking the ITRs in pCAVGFP [10], we inserted complementary oligonucleotides containing the 18-bp I-SceI site into each site using standard molecular biology techniques (primer 1 NotI-I-SceI: AGCAAAAAC AGGAAGGCAAA; primer 2 NotI-I-SceI: CACCGTGTCA ACCACAAAAC; (primer 3 AscI-I-SceI: AAATCTTCC GCAAACAGTGG; primer 4 AscI-I-SceI: TTTTTGTGAT GCTCGTCAGG). The sequence at the left ITR is …CGCTAGGGATAA ?CAGGGTAATATAGCCTTAATTAAGGCCG-**CATCAT…** and the sequence at the right ITR is **ATGATG-**GCGGCCAGTTACGCTAGGGATAÂCAGGGTAATATAGGCG (CAV-2 ITR in **bold**, I-SceI site underlined, cleavage site denoted by “?”). The anti-HA antibody (HA3f10, Roche) was used at a dilution of 1/300 (v/v) and the secondary (anti-rat Alexa-Fluoro488) at a dilution of 1/100 (v/v).

### DNA Damage Response

Prior to OHTam-induced I-SceI translocation, we incubated the cells with 50 µM carbobenzoxy-valyl-alanyl-aspartyl-[O-methyl]-fluoromethylketone (Z-VAD-FMK, Sigma), 10 µM 2-morpholin-4-YL-6-thianthren-1-YL-pyran-4-one (KU-55933, Sigma), 10 µM *Z*-5-(4-hydroxybenzylidene)-2-imino-1,3-thiazolidin-4-one (mirin, Sigma) [27] or 5 mM 3,7-dihydro-1,3,7-trimethyl-1*H*-purine-2,6-dione (caffeine, Sigma). At each wash or medium change the drug was included. Three days posttransfection we collected the cells and processed them as above and the cleared supernatant assayed for GFP expression.

### Statistical Analyses

Statistical analyses were performed using a Mann Whitney test. Two-tailed P values are reported.

## Results and Discussion

### An E1-transcomplementing, I-SceI Canine Cell Line and Plasmids Harboring I-SceI Recognition Sites

**Figure 1:**
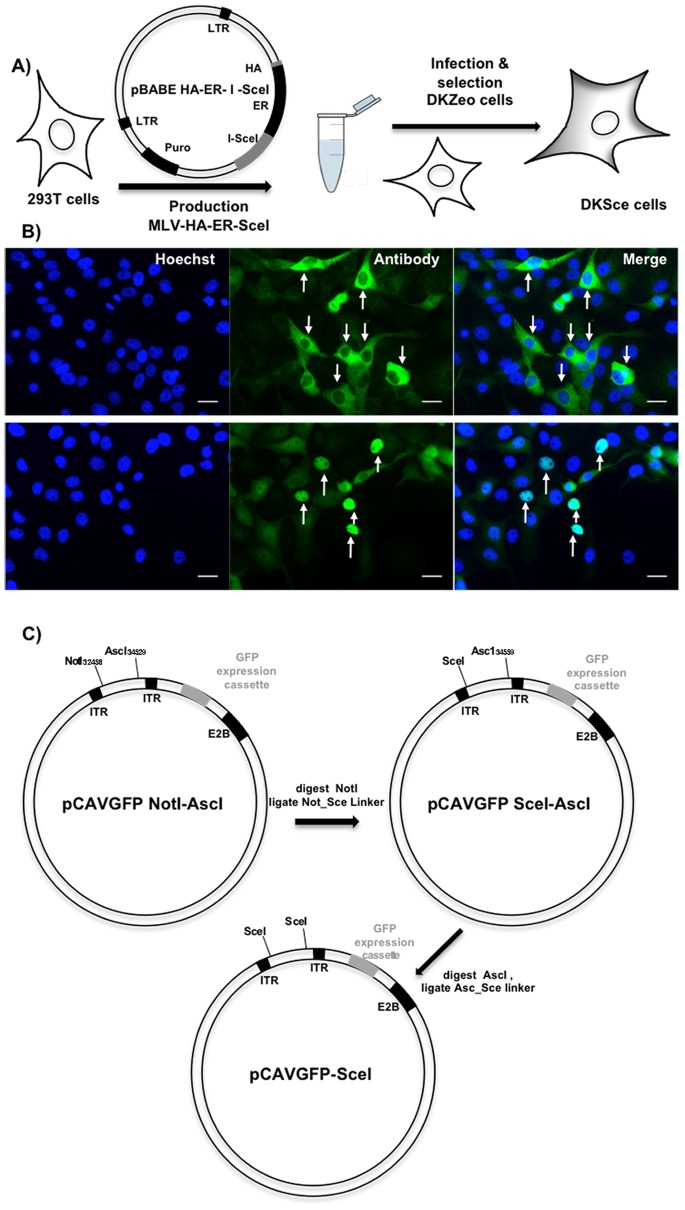
Plasmids, vectors and cells for I-SceI-mediated CAV-2 vector generation. **A) DKSce cells:** A vesicular stomatitis virus g protein (VSVG) pseudo-typed murine leukemia virus (MLV) vector harboring an HA-ER-SceI expression cassette was generated by transfecting 293 cells with pBabe-I-SceI-ER and relevant plasmids The cleared supernatant was incubated with DK CAV-2 E1-transcomplementing cells to generate polyclonal population of DKSce cells via selection by puromycin resistance. **B)** DKSce cells were screened for HA-ER-SceI expression using an anti-HA antibody (in green). In the upper panels the staining is predominantly cytoplasmic (white arrows). DKSce cells were then incubated with OHTam (lower panels) to induce nuclear translocation, where the signal becomes predominantly nuclear (white arrows). Nuclei are stained with Hoechst (blue). Scale bar = 10 µm. **C)** pCAVGFP-AscI-NotI contains a CAV-2 vector harboring a GFP expression cassette replacing the E1 region. It has a unique NotI restriction site downstream of the CAV-2 ITR2 and a unique AscI restriction site upstream of the right CAV-2 ITR. pCAVGFP-SceI was generated by digesting the pCAVGFP NotI-AscI with NotI and ligation with the linker NotI-I-SceI and then by cloning a second I-SceI recognition site upstream of the left CAV-2 ITR.

To improve CAV-2 vector generation we reasoned that the initial hurdle was to improve the transfection efficiency of the canine cells. One option was to identify a cell line that permitted CAV-2 propagation that was also readily transfectable. We screened a handful of canine cell lines, but none was efficiently transduced by linear 30 kb DNA fragments (not shown). However, DK (and MDCK) transfect more efficiently with supercoiled plasmids. If supercoiled plasmids containing vector genomes are transfected, one needs to generate free vector inverted terminal repeats (ITRs) to allow replication [4]. Several approaches are possible to cleave DNA in the mammalian genome: meganucleases, transcription activator-like effector nucleases (TALENS) or zinc-finger nucleases (ZFN). While TALENS and ZFN theoretically allow higher specificity, I-SceI, which is a homing endonucleases that has an ∼18 bp recognition site, has been used by others to facilitate HAdV vector generation [28], [29], [30]. One critical criterion was the possibility of I-SceI cutting the canine genome, inducing apoptosis via the detection of double-stranded DNA breaks, and therefore preventing the selection of a stable cell line that expressed I-SceI. The canine genome does not have I-SceI consensus sites (not shown), but I-SceI, like other homing endonucleases, does not have a stringent recognition sequence and single base pair changes do not abolish cleavage, but rather create variable efficiency. Therefore, to better control I-SceI activity and preclude selecting cell lines with low I-SceI activity, we generated a murine leukemia virus (MLV) vector harboring an estrogen receptor (ER)-I-SceI-hemagglutinin (HA) fusion protein (ER-I-SceI-HA) expression cassette [25] (**Figure 1a**). Fusing ER to I-SceI should maintain I-SceI in the cytoplasm. The addition of 4-OH-tamoxifen (OHTam), an antagonist of the ER, induces the nuclear translocation of ER-I-SceI-HA, where the supercoiled plasmid can be cleaved and replication of the CAV-2 genome initiated. The MLV-ER-I-SceI-HA-infected cells were selected for resistance to puromycin to generate DKSce cells (**Figure 1a**). The resulting polyclonal population was screened for I-SceI expression and translocation using the anti-HA antibody and OHTam (**Figure 1b**). DKSce cells were kept in culture for >10 passages without a detectable loss of ER-I-SceI-HA expression (not shown) consistent with the lack of I-SceI toxicity when fused with ER in canine cells.

Starting from pCAVGFP [10], a plasmid containing a ΔE1 CAV-2 vector with a GFP expression cassette, we generated pCAVGFP-SceI (**Figure 1c**). Using unique NotI and AscI restriction sites immediately flanking each ITR, we inserted complementary oligonucleotides containing the I-SceI site into each site. Control digestions by recombinant I-SceI and agarose gel electrophoresis showed that a 2 kb fragment was released (not shown).

### Transfection of Linear versus Supercoiled DNA in DK Cells

**Figure 2:**
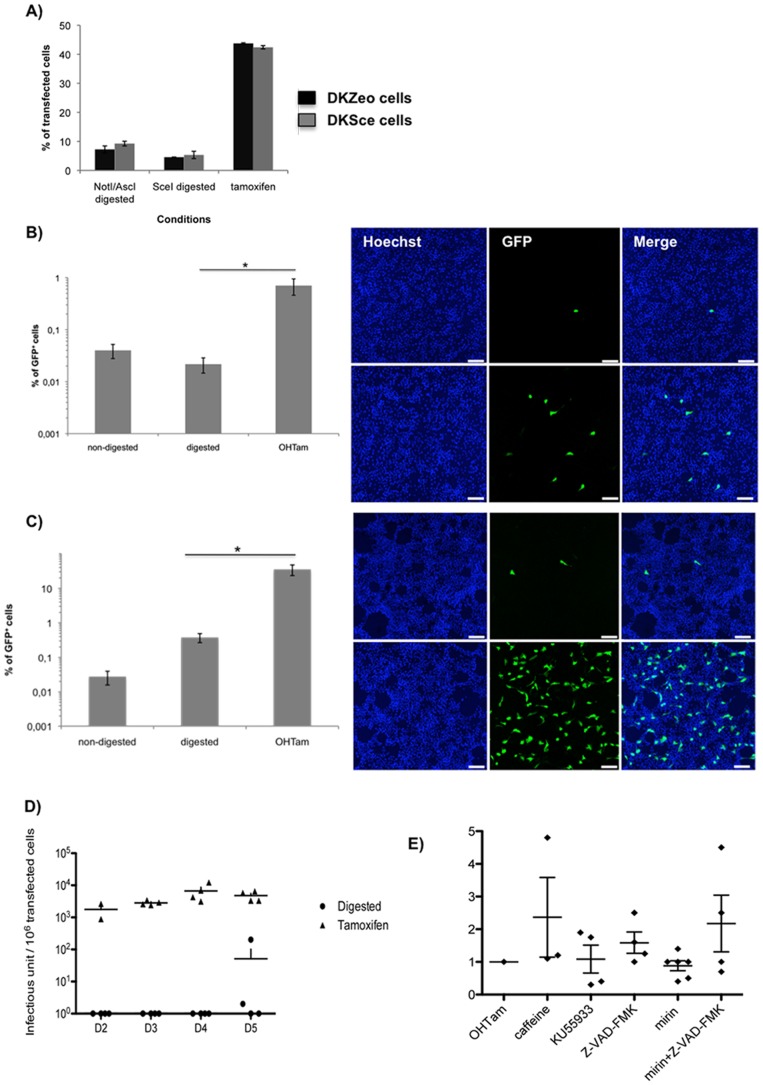
Transfection and vector generation. **A) Transfection of DKE1 cells and DKSce cells with linear or circular DNA.** For each cell line, the level of transfection, based on GFP detection by flow cytometry, was 4–8% for NotI-AscI or I-SceI digested pCAVGFP or pCAVGFP-Sce. Transfection efficiency (±OHTam) increased 7–10 fold when supercoiled 30 kb plasmids were used. **B)** DKSce cells were transfected with supercoiled pCAVGFP-Sce, I-SceI digested pCAVGFP-Sce or supercoiled pCAVGFP-Sce+OHTam. The transfected cells were collected 5 days later, and the cleared lysate used to infect 10-cm plates of DKSce cells. At this vector generation step, GFP^+^ cells were quantified by flow cytometry and by scanning for fluorescence by microscopy. No GFP^+^ cells were detected when transfecting supercoiled pCAVGFP-Sce without OHTam (non-digested). A non-representative image showing rare GFP expression in I-SceI digested pCAVGFP-Sce (digested), and a representative image showing the GFP expression in supercoiled pCAVGFP-Sce+OHTam (OHTam). Nuclei are stained with Hoechst (blue). Scale bar = 10 µm. *P value = 0.005. **C)** A 10-cm plate of DKSce cells was incubated with cleared lysate from the above CAVGFP generation step. No GFP^+^ cells were ever detected in the cells transfected with supercoiled pCAVGFP-Sce (non-digested) and reamplified. Approximately 0.2% of the cells were infected by CAVGFP when using the cleared lysate from cells transfected with I-SceI-digested pCAVGFP-Sce (digested). Greater than 10% of the cells in the 10-cm plate were infected with CAVGFP when using the cleared lysate from cells transfected with supercoiled pCAVGFP-Sce+OHTam Nuclei are stained with Hoechst (blue). Scale bar = 10 µm, *P value = 0.029. **D)** To determine if we could generate vectors more quickly, we repeated the vector generation step using I-SceI digested pCAVGFP-Sce and supercoiled pCAVGFP-Sce+OHTam. The cells were collected at days 2–5 and the cleared lysate was incubated with a fresh monolayer of DKSce cells. The number of CAVGFP infected cells/million transfected cells was quantified. The assays were performed in duplicate and repeated at least three times. **E)** To determine if we could inhibit or modify the DSB break response, and in turn increase CAV-2 vector generation, we included drugs (caffeine, KU55933, Z-VAD-FMK, and mirin) that play a role in preventing DSB recognition, repair or downstream events. Z-VAD-FMK and mirin were also combined. No significant difference was seen versus controls. The assays were performed in duplicate and repeated at least twice.

Over the last 15 years, we tested >60 transfection reagents to improve the transfection of canine cells with linear DNA fragments >30 kb. Why DK cells are less transfectable than other cell lines is unknown. Reduced transfectability of linear DNA fragments could be due to larger DNA-transfection reagent complexes and/or the presence of exonucleases that degrade free DNA ends. Currently, our optimized conditions using 2 µl of Turbofect/µg of DNA leads to efficacies of 5–7%. Using these conditions, transfection of supercoiled plasmids <10 kb can reach >70% efficiency, but transfections using plasmids >30 kb was near 50% (**Figure 2a**). While still not as efficient as in 293 cells (where greater than 90% efficacy is possible), the 7 to 10-fold increase in the number of cells, in combinations with more genomes/transfected cell, could skirt the CAV-2 vector generation bottleneck.

We then compared our standard protocol for CAV-2 vector generation to the combination of DKSce cells transfected with supercoiled pCAVGFP-SceI/OHTam (I-SceI-OHTam approach). DKSce cells were transfected with the circularized or linearized plasmids, the medium was replaced with fresh medium and the cells were kept at 37°C/5% CO_2_ for 5 days. Then the cells were collected, and cleared lysate incubated with a fresh monolayer of cells for 24 h. The plates were then screened by fluorescent microscopy, and by flow cytometry. Similar to the results found with plasmid HAdV5 vector genomes [4], circular pCAVGFP-SceI did not lead to vector generation (**Figure 2b**). I-SceI linearized pCAVGFP-SceI and AscI/Not1 linearized pCAVGFP produced similar amount of CAVGFP, demonstrating that in this case there was no difference in CAVGFP generation due, for example, to the number of nucleotides remaining after the ITRs [4]. In each of these approaches, we routinely identified <20 GFP+ cells by fluorescent microscopy in a 10-cm plate, and only in rare 1 cm^2^ visual fields was a GFP^+^ cell detected (**Figure 2b** **, top panel**). As expected, when we assayed these cultures by flow cytometry, the level of background (0.05%) did not allow us to detect a significant increase in GFP^+^ cells versus mock-treated cells.

By contrast, when using the above condition with the I-SceI-OHTam approach, we obtained >1% GFP^+^ cells in a 10-cm plate containing 5×10^6^ DKSce cells (i.e. 5×10^4^ GFP^+^ cells). This difference (<20 GFP^+^ cells compared to 50,000 GFP^+^ cells) corresponded to a 1,000-fold increase in CAV-2 vector titre. This significant increase in efficacy was also prominent at a second amplification when control and I-SceI-OHTam cultures were collected, lysed and incubated with fresh cells (**Figure 2c**). Again, we screened the cultures by fluorescent microscopy and flow cytometry. Visual fields containing GFP^+^ were more abundant and detection by flow cytometry was above background using the previous protocol, however there was a 200-fold increase in GFP^+^ using the I-SceI-OHTam approach.

To determine if the I-SceI-OHTam approach could also decrease the time of CAV-2 vector production, we repeated the above protocol and lysed the cells at 2, 3, 4 and 5 days posttransfection (**Figure 2d**). As per our previous experience, generating CAVGFP when linear pCAVGFP was transfected, collected and lysed at 2–4 days was, bar a few exceptions at day 4, unproductive. By contrast, the I-SceI-OHTam approach generate >1,000 infectious particles/10^6^ transfected cells as early as day 2 (**Figure 2d**). We then tested the I-SceI-OHTam approach by generating a CAV-2 vector harboring a channelrhodopsin expression cassette – a vector that we could not produce via the previous method. Channelrhodopsin overexpression is thought to perturb endoplasmic reticulum (ER) function [31]. In spite of this toxic effect, we succeeded in producing this vector demonstrating the efficacy of the I-SceI-OHTam approach (not shown).

### Dampening Double-stranded DNA Break (DSB)-induced Response

We noticed that 24 h posttransfection with pCAVGFP-SceI and OHTam-induced translocation of I-SceI, the DKSce cultures degraded with the classic appearance of apoptotic cells. Because this effect was not seen with OHTam or transfections alone, we speculated that the effect was due to the amount of free DNA ends generated by I-SceI cleavage of the transfected plasmid, and therefore the initiation of DSB-induced apoptosis [32]. It was likely this effect was not detected in cells transfected by linear fragments because of the poor transfection efficiency and the paucity of nuclear DNA fragments compared to supercoiled plasmids.

Under most physiological conditions (e.g. during replication & meiosis, immunoglobulin or T cell receptor genes rearrangements), DSB are repaired by HR and nonhomologous end joining (NHEJ) recombination. Recognition of lesions starts a cascade, which results in cell cycle arrest (checkpoint activation) and DNA repair. If DNA repair fails, or is overwhelmed, cells undergo death by activating apoptosis [32]. Therefore, in the I-SceI-OHTam approach, we tried to dampen apoptosis long enough to allow CAV-2 vector propagation. We tested the effect of Z-VAD-FMK, KU-55933, mirin and caffeine, which inhibit different stages of the DNA damage response. Z-VAD-FMK is a pan-caspase inhibitor that binds their catalytic sites and can inhibit induction of apoptosis [33]. KU-55933 inhibits DNA-PK and PI3K, prevents the activity of mTOR and ablates protein kinase ataxia-telangiectasia mutated (ATM)-dependent phosphorylation, which is activated in response to DNA damage [34]. Caffeine, a xanthine derivative, leads to inhibition of the G_1_, intra-S, and G_2_ cell cycle checkpoint by blocking ATM kinase activity and phosphorylation of cyclinB [35].

Together with OHTam-induced-I-SceI translocation, we incubated the cells with 50 µM Z-VAD-FMK, 10 µM KU-55933, or 5 mM caffeine. Three days posttransfection we collected the cells and processed them as above and tested the cleared supernatant for CAVGFP production. Although in some assays outliers suggested a positive effect, we found no significant or reproducible improvement in CAV-2 vector production (**Figure 2E**). Only Z-VAD-FMK produced consistent and modest increases in CAVGFP production, but it did not reach statistical significance (*p*>0.05). Of note, Z-VAD-FMK also inhibited the appearance of an apoptotic response (not shown).

Another probable mechanism inhibiting CAV-2 vector production is DSB repair (DSBR). The downstream signalling from DSBs requires the interplay between the Mre11/Rad50/Nbs1 (MRN) complex and ATM [36], [37]. Depending on the quantity, speed and efficacy of I-SceI, HR and NHEJ likely act at early phases. But as I-SceI cleaves all available sites, NHEJ should be the primary mechanism for repair and in its error-prone manner ligate the DSB ends. Following DSB formation, MRN complex recognizes the free ends [32]. Mre11 has exo- and endonuclease activity, Rad50 has DNA binding capabilities and Nbs1 shuttles the complex to the nucleus. Rad50 holds the DSB ends together while Mre11’s nuclease activity processes the DSB ends. Once the MRN complex is engaged, ATM is recruited. In the I-SceI-OHTam approach, CAVGFP concatemers could be formed by NHEJ, which may be amplified by errors in the I-SceI recognition sites that prevent I-SceI re-cleavage. Notably, this phenomenon is critical during the initial stages of vector generation because the two mechanisms that adenoviruses use to circumvent the DNA damage response (E4-Orf3-dependent relocation of MRN proteins and E4-Orf6/E1B-55K-dependent degradation of MRN components [38], [39]) are not functional yet. We therefore tested the effect of mirin, which prevents MRN-dependent activation of ATM without affecting ATM protein kinase activity and it inhibits Mre11-associated exonuclease activity [40], [41]. Mirin abolishes the G2/M checkpoint and homology-dependent and homology-independent repair in mammalian cells. Like above though, the effect of mirin did not reach statistical significance (*p*>0.05) (**Figure 2E**). Because under some conditions we saw a trend towards improved CAVGFP generation, we combined Z-VAD-FMK and mirin, two drugs with complementary mechanisms of action. However, we were not able to further increase CAV-2 vector titres significantly beyond the 1,000-fold increase found with the I-SceI-OHTam approach.

### Conclusion

In our hands, HAd5V vector generation is very efficient (>95% success after the initial transfection), while CAV-2 vector generation is successful in <50% of our attempts. In this study we improved CAV-2 vector titres posttransduction by 1,000 fold, and in turn increased the efficacy of vector generation. In addition, we decreased the time of vector generation by two weeks when taking into account the generation at day 2 instead of day 5, and the two or three extra amplification steps (each 2 days) needed to produce the same amount of vector posttransfection. These technical advances will facilitate the generation and use of CAV-2 vectors by laboratories with and without significant expertise in cell culture and molecular biology.
